# Towards a sane and rational approach to management of Influenza H1N1 2009

**DOI:** 10.1186/1743-422X-6-51

**Published:** 2009-05-07

**Authors:** William R Gallaher

**Affiliations:** 1 Department of Microbiology, Immunology and Parasitology, Louisiana State University Health Sciences Center, 1901 Perdido Street, New Orleans, Louisiana 70112, USA

## Abstract

Beginning in March 2009, an outbreak of influenza in North America was found to be caused by a new strain of influenza virus, designated Influenza H1N1 2009, which is a reassortant of swine, avian and human influenza viruses. Over a thousand total cases were identified with the first month, chiefly in the United States and Mexico, but also involving several European countries. Actions concerning Influenza H1N1 2009 need to be based on fact and science, following recommendations of public health officials, and not fueled by political, legal or other interests. Every influenza outbreak or pandemic is unique, so the facts of each one must be studied before an appropriate response can be developed. While reports are preliminary, through the first 4 weeks of the outbreak it does not appear to be severe either in terms of the attack rate in communities or in the virulence of the virus itself. However, there are significant changes in both the hemagglutinin and neuraminidase proteins of the new virus, 27.2% and 18.2% of the amino acid sequence, from prior H1N1 isolates in 2008 and the current vaccine. Such a degree of change qualifies as an "antigenic shift", even while the virus remains in the H1N1 family of influenza viruses, and may give influenza H1N1 2009 significant pandemic potential. Perhaps balancing this shift, the novel virus retains more of the core influenza proteins from animal strains than successful human influenza viruses, and may be inhibited from its maximum potential until further reassortment or mutation better adapts it to multiplication in humans. While contact and respiratory precautions such as frequent handwashing will slow the virus through the human population, it is likely that development of a new influenza vaccine tailored to this novel Influenza H1N1 2009 strain will be essential to blunt its ultimate pandemic impact.

## Introduction

On April 9, 2009 it became apparent to public health officials in Mexico City that an outbreak of influenza was in progress late in the influenza season [1]. On April 17, two cases in children were also reported in California near the Mexican border [2]. Virus samples were obtained and the virus determined to be a novel strain of influenza A of the H1N1 serotype. Preliminary tests conducted by the Centers for Disease Control and Prevention (CDC) indicated that the virus was a novel reassortant, containing genetic elements of influenza viruses found in swine, birds and human beings.

Influenza virus, an enveloped virus of the *Orthomyxoviridae *family, has a unique capacity for genetic variation that is based in two molecular features of the virus family [3]. First of all, the surface proteins of the virus are highly variable, able to mutate up to 50% of their amino acid sequence and still perform their functions in infection. Secondly, the viral genome is segmented, with eight RNA segments that are genetically independent of one another. In a mixed infection of different influenza genotypes, these segments can almost randomly reassort resulting in hybrid genotypes with some segments derived from one virus strain, while the other segments are derived from a second strain.

Less than one month later, hundreds of probable cases of infection by this novel virus, designated Influenza H1N1 2009, had been identified, with 26 deaths, centered about the area of Mexico City. An additional several hundred probable cases had been identified in the United States [4], most associated with recent travel to Mexico, and concentrated in California, Texas and New York. Sporadic cases, also associated with travel to Mexico in large part, were found in several European countries as well. The World Health Organization (WHO) began to declare ever higher stages on its "pandemic" scale, designating the novel Influenza H1N1 2009 a potential threat to worldwide health [5]. Press coverage and involvement of public officials in the response to the novel virus has reached epic proportions.

This commentary is intended to review and analyze the salient facts of the outbreak and the molecular sequence of the principal external antigens of Influenza H1N1 2009. The discussion will focus on the implications of this analysis for the continued course of the outbreak and the medical response.

## Discussion

### Tenor of the Response to Influenza H1N1 2009

Actions concerning Flu H1N1 2009 need to be based on fact and science, following recommendations of public health officials, and not fueled by political, legal or media interests and hysteria. This is time for calm, thoughtful action, and not the panic we have seen spread around the globe inspired by media reports. When 10 schools or an entire school district are closed due to one suspected case of influenza, we might well ask if our response has been measured and appropriate. The good faith of the public is a precious commodity. When one day a pandemic is trumpeted, and the next day the outbreak is called no more than normal flu and under control, and then a call goes out for a multibillion dollar vaccine program to defend against a major pandemic, one risks the public feeling whiplash and the credibility of public officials being damaged. Further, every measure of response has a cost-benefit ratio that needs to be carefully considered, which is best done in collaboration with public health professionals. We have seen unnecessary and useless quarantines, interdictions of trade and excessive closures which cannot be sustained and have little if any benefit. Travel in and out of Mexico has been severely disrupted, but not to New York City which also has many confirmed cases. A cruise ship plies the Pacific, avoiding Mexican ports with little or no influenza activity, but plans to host its passengers an extra night in San Diego, with a higher number of H1N1 cases in the area than most areas of Mexico. At some point in what will probably be a long engagement with this new influenza strain, a more precisely targeted and rational response will be needed.

### The Enigma of Response and Responsibility

Every influenza outbreak or pandemic is unique, so the facts of each one must be studied before an appropriate response can be developed [3]. No actual pandemic matches the theoretical influenza pandemic or past history. Each must be judged on its own evolution. The only really accurate assessments have been retrospective, after years or decades of further analysis, so it is important for both the scientific and general public to understand that decisions will need to be made using the best information available at the time and will be fallible. There can be no standard playbook. However, fallible does not mean irrational. Even though elected and corporate officials are charged with the responsibility to make such decisions, and no one wishes to be found negligent in retrospect, the best course is to closely follow the recommendations of recognized experts in the field of influenza virology and public health who have made the study and understanding of this viral disease their life's work. The WHO, CDC, academic virologists and physicians, and state epidemiologists know their business and should be trusted to guide public policy. An elected official cannot and should not try to reproduce and override, with an hour's briefing, their cumulative decades of experience. This is no time to haul out tired agendas concerning immunization or immigration or cultural and ethnic biases, using influenza for cover.

### Nature of the Outbreak to Date

This virus constitutes a serious threat not based on the outbreak thus far, which has been, in historical terms, very limited in the total number of probable cases, but rather on the potential of the virus. To date, influenza H1N1 2009 has not made a very successful penetrance into the human population. Even if 22,000 in Mexico City were infected, a high estimate, it would constitute only 0.1% of the population of 22 million – one of the more populous metropolitan areas on earth. In contrast, in a "normal" influenza season, with an "ordinary" strain of influenza, there are 200,000 cases and 36,000 deaths in only a few months each winter in the United States alone [6]. Classic pandemic flu attack rates are, unfortunately, far higher. Indeed, influenza is in a class of its own for its potential ability to infect enormous numbers of humans in a very short period of time – thus far with H1N1 2009 we are not even close to that level. This may technically be a "pandemic" in the sense of human-to-human virus transmission of a novel virus in more than one region of the planet, but would not yet be recognizable as an influenza pandemic to anyone who has lived through one.

There are two very separate meanings for "severity" when discussing influenza. One relates to the virulence of the virus in any given host; the other to the attack rate, or numbers of cases of infection per unit of population. Thus, one can have a "severe" pandemic, affecting millions of human beings, with a relatively avirulent – not severe – influenza virus that results in relatively few hospitalizations or deaths. On the other hand, one can have a limited outbreak, such as Southeast Asia has experienced in recent years with bird flu, with a highly virulent, severe virus that produces very high percentages of hospitalization and high mortality.

While it is very early to properly evaluate public health reports and statistics, the Influenza H1N1 2009 outbreak thus far does not appear to be severe in either respect. However, within the viral genetic sequence there is at least the potential for a severe pandemic. Also, an unusually high number of cases appear to be in previously healthy young adults, a feature found more commonly in the more virulent influenza viruses.

Since influenza was first isolated in the 1930s, it has been axiomatic that the severity of an epidemic or pandemic is proportional to the susceptibility of the human population, which is in turn directly related to the degree of change in the surface proteins of the virus, the H and N antigens [7]. The greater the change, the less that preexisting human antibodies to influenza can neutralize the virus, and the lower the "herd immunity" of the entire human population. Minor incremental changes in these antigens, denoted as "antigenic drift", lead to mild outbreaks. Major, sudden changes in these antigens, denoted as "antigenic shift", have led to the major pandemics of influenza in the 20th century. There has not been a major antigenic shift in human influenza since 1968.

### Changes in the Hemagglutinin

The major component of influenza virus that determines its epidemiological dynamic is the predominant surface protein on the viral envelope, the H antigen. This protein serves as the hemagglutinin or HA1 attachment protein. It determines whether the virus is able to bind to and infect cells of different species by its ability to attach to carbohydrate receptors on the cells. The protein loops that determine the sites of binding for antibody dominate the immune response to the virus. Thus the H antigen is the principal component of any influenza vaccine and the efficacy of the vaccine is measurable by determining the ability of the elicited antibodies to neutralize viral binding.

**Figure 1:**
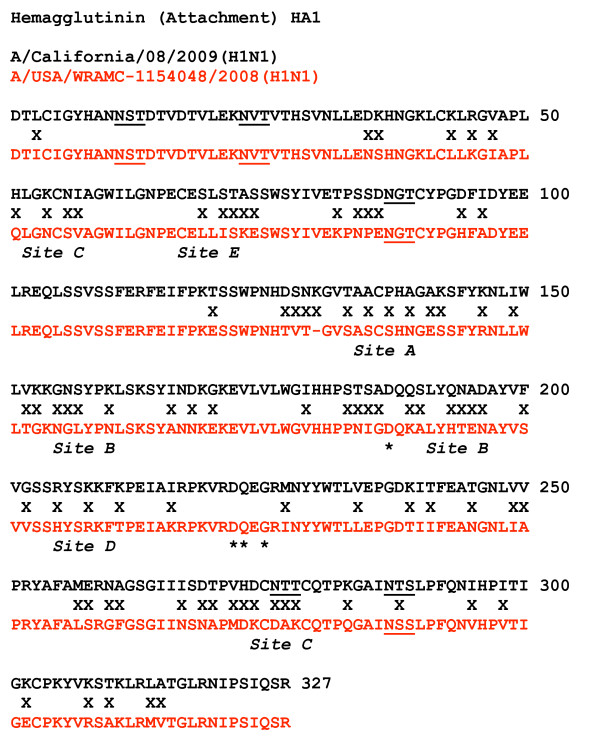
**Amino acid sequence alignment of the mature hemagglutinin (H) proteins of Influenza H1N1 2009 and its predecessor H1N1 isolated in 2008.** The amino acid sequence of the H protein shown for the Influenza H1N1 2009 virus is derived from the segment 4 sequence of the isolate A/California/08/2009(H1N1) submitted from the CDC by Shu et al. on April 29, 2009, as Genbank FJ971076.  The sequence for Influenza H1N1 2008 is derived from the segment 4 sequence of the isolate A/District of Columbia/WRAMC-1154048/2008(H1N1) submitted from Walter Reed Army Institute of Research by Houng et al., collected from a patient on February 1, 2008, as Genbank CY038770. Only the sequences of the mature proteins, after cleavage of the signal sequence, are shown. Standard single-letter abbreviations for the amino acids are used. The collinear sequences were hand-aligned and also confirmed by online use of ClustalW. Amino acid positions showing differences between the two sequences are denoted with an “X”. There are 89 differences in 327 positions, or 27.2%. The canonical sites for N-linked glycosylation are  underlined. Amino acid regions contributing to each of five antigenic sites are labeled Site A through Site E.

Figure 1 shows an amino acid sequence alignment of Influenza H1N1 2009 with its predecessor HIN1 virus isolated the previous year, in 2008, at Walter Reed Army Hospital in Washington, DC. (The 2008 virus is in turn identical in amino acid sequence to the H antigen in the current influenza virus vaccine.) Each change in the sequence of Influenza H1N1 2009 from the 2008 virus is marked with an "X" in the alignment. It is obvious that H1N1 2009 is significantly novel, 27.2% different from the human H1N1 virus circulating in 2008 and the H antigen in the current vaccine. Also noted in the figure are the canonical sites for N-linked glycosylation of the protein, at NxS/T motifs (underlined), as well as the approximate positions of amino acids that determine the antigen specificity at five different protein loop regions on the surface of the protein, designated Site A through Site E [8]. It is obvious that the changes in amino acid sequence are concentrated in these antigenic sites. Additionally, one of the sites, Site C, may be blocked by a novel N-linked glycosylation at N277. All five of the known antigenic sites on the protein are therefore unique, and so no human herd immunity to this virus is to be expected anywhere in the human population of 6.77 billion persons. This constitutes a major antigenic shift which has in the past been the basis of major human pandemics.

Additional sequence comparison (not shown) indicates that, as stated by others in the press several times, Influenza H1N1 2009 is not similar to the 1918 pandemic influenza virus (18% different), and not similar to the 1976 swine flu from Ft. Dix, New Jersey (12% different). Also, the amino acids most critical in specifying receptor usage [9], indicated in the sequence alignment by asterisks, are identical to current human H1N1. Thus the spectrum of human infection in the respiratory tract is not likely to be unusual relative to the 2008 H1N1 or other recent influenza strains. These are positive features of the virus arguing for a lower level of virulence.

### Changes in the Neuraminidase

The second external protein of influenza virus, constituting 20–25% of the surface proteins, is the N antigen. This protein is an enzyme named neuraminidase for its ability to cleave neuraminic or sialic acid from complex carbohydrates such as mucins. In infection it serves to allow release of newly produced virus from surface receptors and to digest mucous secretions, allowing the virus better access to the surface of susceptible cells and spread through the respiratory tract. Its value as a spreading factor is underscored by the fact that the currently licensed antiviral drugs oseltamivir (Tamiflu) and zanamivir (Relenza) function as neuraminidase inhibitors. In the absence of herd immunity to the H antigen, partial protection can be provided if the same or similar N antigen is retained. Eickhoff and Meiklejohn showed that the infection rate with the H3N2 virus was reduced up to 50% in Air Force cadets who had received the H2N2 vaccine, due to the shared N2 antigen remaining identical [10]. If the N1 antigen of the 2009 virus proved to be similar to that of 2008, even with an antigenic shift in H, then some cross protection from prior H1N1 infection or the 2008 vaccine might be expected. Unfortunately, in the case of influenza H1N1 2009, the N1 antigen also is significantly novel, differing by 18.2% from the 2008 H1N1 virus. While the antigenic sites within the N antigen are less well defined (reviewed in [11]), the pattern of changes in the N antigen of the 2009 virus (not shown) are not encouraging. No cross protection is likely.

### Implications from Sequence Changes in H1N1 2009

Overall, it is clear from the sequence alignments of the Influenza H1N1 2009 virus that, even though this virus is still basically in the family of H1N1 viruses, the sequence changes indicate a significant antigenic shift in both surface antigens. The last time such an antigenic shift occurred in both H and N antigens was the 1957 Asian H2N2 pandemic.

A factor present in 1957 was that there was serological evidence that those over 60 years of age retained an anti-H2N2 antibody response from prior exposure to the virus before 1900 [12]. This blunted the effect of the 1957 pandemic in the elderly. This factor is not expected in the case of H1N1 2009, since there is no evidence that a virus with a similar antigenic profile has circulated in the human population in over 100 years.

### Neither Swine Nor Mexico Are to Blame

The outbreak is due to a rare recombination of influenza gene segments from swine with avian and human influenza. Once this one time event occurred, swine are not a significant immediate source of the human version of influenza H1N1 2009, and the virus cannot be acquired from handling or eating pork. The consensus among virologists is that the actual natural host and ultimate source of influenza variants is migratory waterfowl. [13]. The prospective slaughter of pigs in Egypt, and the international interdiction of imported pork, have no rational basis in science or public health.

As for this being a "Mexican" virus, analysis of the H sequence by BLAST [14] reveals that the closest relative to the Influenza H1N1 2009 virus previously isolated is in fact a virus 95% identical to it, from swine in Indiana in 2000 (e.g. A/Swine/Indiana/P12439/00 (H1N2)). Border interdiction makes no sense when the H gene is All-American, having been in Indiana longer than the Head Coach and most of those playing football for Notre Dame. Similarly, the closest neuramindase sequence, 94% identical, is one isolated in Britain and elsewhere in Europe in the 1990s (e.g. A/Swine/England/195852/92 (H1N1)). The parts of the virus may well have been imported into Mexico, and accidentally assembled the new influenza 2009 virus there, leading to emergence by pure happenstance. Such emergence can happen anywhere. Retrospective analysis revealed that the 1918 H1N1 virus, dubbed the "Spanish" flu for decades, is likely to have arisen in the United States [15]. Assigning blame or even a country of origin for an emergent virus is a dubious exercise more likely to reinforce cultural bias and prejudice, and ignite non-cooperation, than to be helpful in controlling influenza.

### Factors Predisposing to Control of Influenza H1N1 2009

Two additional facts concerning the virus are positive. First, while the most successful pandemic influenza viruses have changed only the H and N antigens and retained the same human core proteins of the virus, influenza H1N1 2009 has several more components from animal flu strains than the H2N2 and H3N2 viruses of 1957 and 1968, respectively. This may make the 2009 virus less compatible with effective replication in humans, which may in turn be holding it back in its penetrance of the human population. Second, the 2009 virus is sensitive to the two neuraminidase inhibitors licensed as antiviral drugs. A reasonable conclusion from these last two facts is that there is no evidence at all that this is a bioterror event, but rather a novel virus perpetrated by nature alone.

### Immediate Prospects for Control

The outbreak appears to be waning or controlled at its origins and certainly not growing logarithmically or of truly pandemic proportions. However, influenza exhibits marked seasonal occurrence even in pandemic years. We have reached the end of the classic flu season in the Northern Hemisphere, and not yet begun that season in the Southern Hemisphere. The outbreak could wane even if we were not doing everything right; indeed it could wane even if we were doing everything wrong, simply because that is what the flu does this time of year. Its true potential may not be revealed until the onset of the flu season in the Northern Hemisphere in October or November of 2009.

### Further Evolution of the Virus Possible

The greatest instability of a novel human virus is when it first enters the human population and is under very heavy selective pressure in the environment of the human respiratory tract. As the influenza season in the Northern Hemisphere ends, the virus could simmer for months and co-circulate with the 2008 strains of influenza. While recombination events in the human population have not been documented, the virus could shed more genes from its animal sources, acquire more human influenza genes and become better adapted to human replication and spread. A virus with the new coat of H and N antigens, built onto the core of prior successful human pandemic influenza viruses, could be a threat exceeding anything we have seen since 1918, given the great increase in human populations over the last 50 years. Further reassortment of viral genes in pigs are also possible [16]. Alternately, incremental mutations in other genes of the virus may achieve the same result of enhanced replication in humans without further recombination. There is simply no way to tell where H1N1 2009 will evolve. The only honest answer to the question of how this outbreak will evolve over the next 6 to 18 months is: "I don't know."

### General Planning for a Long-Term Response

It is for the continued circulation of an enhanced H1N1 2009 that we should plan, and develop a vaccine based on the novel H and N antigens. Given only a few months, and a worldwide capacity of only about 500 million doses of human vaccine using present methods, use of vaccine and antivirals must be rational and carefully controlled. Already there is evidence that Tamiflu is in high demand disproportional to actual cases, indicating possible attempts to either use the drug for prophylaxis or to horde it for later use. If true pandemic attack rates are reached later this year or next, there is risk that medical professionals would lose control of a valuable resource to treat the ill. Recall that when only a few individuals received letters laced with anthrax, the antibiotic ciprofloxacin became scarce very quickly. Measures need to be taken to assure that a similar scenario is not possible with the limited amount of antivirals available.

Common sense preventive measures, such as frequent handwashing and discretion on close personal human contact, and carefully targeted school and worksite closures, will buy time and slow down the outbreak, until an effective multi-year vaccine program can provide the best prevention. While influenza virus can survive on inanimate surfaces, it is spread most easily by direct human contact. Contact control among human beings, maintaining literally an arm's length from others wherever practicable, and staying home when sick, will achieve more than all the antiseptic wipes and face masks that can be manufactured. The CDC and WHO are actively promulgating behavioral changes that can reduce the circulation of influenza [17]

Further education and preparation of health care workers and first responders to deal with an influenza pandemic is critical. Only a physician over 60 years of age was even in medical school when the last and mildest influenza pandemic took place in 1968. Only a physician over 70 was in medical school during the last pandemic when both the H and N antigens exhibited significant change, with massive morbidity worldwide in 1957. Few working in virology or the health field today know an influenza pandemic except through the eyes of a child. If and when it happens, it will change our entire frame of reference for epidemic respiratory disease.

### Future Strategies

There is also need for enhanced influenza research and development. The priority of influenza waned in the absence of a pandemic, coupled with the availability of drugs and what seemed to be adequate vaccine technology. However, the antivirals will never have been used to the extent that is likely should this H1N1 2009 outbreak continue. If resistance to these antivirals were to develop due to their overuse and misuse, much as in the case of antibiotics for bacteria, then there is currently no backup drug to combat the virus. Antivirals that inhibit infection and fusion have been developed for viruses such as human immunodeficiency virus (HIV) [18] that have very similar entry mechanisms, and should be developed for influenza as well.

As for the influenza vaccine, it is still produced by relatively archaic methods developed in the 1930s to 1950s using mass quantities of embryonated chicken eggs. We are not far beyond the pioneering days of Goodpasture, Woodruff, Buddingh and Francis in this regard [19]. Each dose of flu vaccine requires the use of 1.2 live eggs, or about 600 million embryonated eggs to produce 500 million does of virus for 6.77 billion people. The math is not encouraging. Vaccines targeting viruses such as measles, mumps, rubella and hepatitis B employ cell culture or recombinant technologies and have superior safety characteristics. Programs for greater efficiency in producing effective and safe influenza vaccines have been too long delayed in development and need to be implemented quickly, to assure that this and future threats of pandemic influenza can be met.

Over the long run, immunization provides the best preventive strategy against influenza virus. Critics revel in citing the 1976 swine flu vaccine, which produced 25 vaccine-associated deaths due to Guillain-Barre syndrome while the virus itself only resulted in one death at Ft. Dix, New Jersey. However, such vaccine-bashing ignores the fact that this fatal complication occurred in only 1 in a million vaccinees, and was not seen either before or since that immunization campaign [3]. Many hundreds of millions of doses of trivalent H1, H3 and B influenza vaccine have been administered over the intervening 30 years without significant complications, while saving countless lives.

As a patient with significant cardiopulmonary disability, I have had clinical influenza three times in my life, in 1948, 1965 and 1974, and been hospitalized twice with secondary pneumococcal pneumonia. Since 1977 I have been routinely administered the influenza vaccine, and not only have I been free of influenza since then, but have twice nursed a spouse to health through influenza. To those critics of influenza immunization I can only say that I am certain that I would choose immunization over the disease, even at the risk of complications or the rare possibility of a vaccine-associated death. To be frank, when I look at the changes in the H1N1 2009 hemagglutinin from the 2008 virus, I see in them the face of my possible executioner. If they need someone to be first in line to receive the new H1N1 2009 vaccine, I hereby volunteer.

Overall, development of antiviral immunizations have long been recognized as the most cost efficient use of public dollars in the entire health field, both in lives saved and economic impact.

## Conclusion

Influenza H1N1 2009 is a novel virus quite unlike even the other H1N1 influenza viruses that have preceded it as agents of human influenza. The fact that its hemagglutinin is 27.2% different and its neuraminidase is 18.2% different in amino acid sequence from the 2008 H1N1 and vaccine virus strains give Influenza H1N1 2009 significant pandemic potential, based on historical pandemics of the 20^th ^century. However, it has yet to prove that potential in what is an outbreak with low community attack rates and modest virulence. Further evolution of the virus toward a more efficient agent of human disease may yet enable it to produce a major pandemic. The future course of the outbreak cannot be predicted, but prudence dictates that a new influenza vaccine, targeted to the novel influenza H1N1 2009 sequence be quickly developed and prepared for worldwide administration. In the absence of existing human "herd" immunity to this virus, only immunization provides a significant hope of suppressing the long-term impact of this newly emergent virus.

## Competing interests

None. The author is retired as a Professor Emeritus from his academic institution, and is not on contract with any academic or corporate entity, nor does he hold a financial interest in any entity deriving profit from any of the pharmaceuticals mentioned in the commentary. He holds one US Patent for an antiviral strategy against Ebola virus, which is unrelated.
